# Fasting: How to Guide

**DOI:** 10.3390/nu13051570

**Published:** 2021-05-07

**Authors:** Alda Attinà, Claudia Leggeri, Rita Paroni, Francesca Pivari, Michele Dei Cas, Alessandra Mingione, Maria Dri, Marco Marchetti, Laura Di Renzo

**Affiliations:** 1 School of Specialization in Food Sciences, University of Rome Tor Vergata, Via Montpellier 1, 00133 Rome, Italy; alda.attina@gmail.com (A.A.); claudialeggeri@gmail.com (C.L.); 2 Department of Health Sciences, University of Milan, Via A. di Rudinì, 8, 20142 Milan, Italy; michele.deicas@unimi.it (M.D.C.); alessandra.mingione@unimi.it (A.M.); 3 PhD School of Applied Medical-Surgical Sciences, University of Rome Tor Vergata, Via Montpellier 1, 00133 Rome, Italy; DRImarystenr@hotmail.it (M.D.); marco@marcomarchetti.it (M.M.); 4 Section of Clinical Nutrition and Nutrigenomic, Department of Biomedicine and Prevention, University of Tor Vergata, Via Montpellier 1, 00133 Rome, Italy; laura.di.renzo@uniroma2.it; 5 Food Working Group, Italian University Network for Sustainable Development (RUS), University of Tor Vergata, Via Cracovia, 00133 Rome, Italy

**Keywords:** fasting, starvation, caloric restriction, personalized nutrition, longevity, body composition, diet, Mediterranean model, anti-inflammatory diet

## Abstract

Fasting potentials are the most interesting topics in the Nutritional Era. Fasting consists of the catabolism of lipids, proteins, and carbohydrates to maintain blood glucose levels in a normal range. The action mechanisms of fasting were firstly understood in minor organisms and later in humans. Nutritional interventions of caloric restriction could attenuate age-associated epigenetic alterations and could have a protective effect against cellular alterations, promoting longevity and health span. While most fasting studies point out the weight and fat mass decreases, it is important to define specific guidelines for fasting and non-fasting days to enhance adherence, minimize the dropout rates of the interventions, and maximize body composition improvement. Although the panorama of evidence on fasting and caloric restriction is wide, there is a lack of a safe fasting protocol to guide physicians in its prescription. The main goal is to identify a how to use guide, a major posology of fasting, inserted within a huge dietetic personalized strategy leading to an optimal and healthy nutritional status.

## 1. Introduction

Recently, fasting has become one of the most compelling topics of the Nutrition Era. In the last five years, interest has passed from the Mediterranean to the Ketogenic Era, including the concept of caloric restriction and ‘only water’ fasting. Fasting is defined as the voluntary abstinence of some or all foods and beverages for therapeutic, spiritual, or political reasons. Recently, research in animal models and humans has highlighted the potential health-promoting physiological responses to fasting including ketogenesis, hormone modulation, reduced oxidative stress and inflammation, and increased stress resistance, lipolysis, and autophagy [1,2].

Although the panorama of evidence on fasting and caloric restriction is wide, there is a lack of a correct and safe fasting protocol to guide Nutritionists and Physicians in its application. The main goal of this article is to identify a how to use guide which may be used in clinical practice and as a nutritional intervention inserted within a huge dietetic personalized strategy.

### 1.1. Historical Background

Fasting is a historical practice that has deep roots in the Christian period. St. Catherine of Siena was one of the early historic cases of extreme starvation. Her diet was characterized by a self-induced restricted regimen composed of only water and vegetables. This was one of the first forms of holy fasting, patently motivated by deep religious belief. This condition, classified as anorexia mirabilis, is also known as inedia prodigiosa [3]. The first modern description of a severe eating disorder occurs 1868 by Sir William Gull. In a lecture at the University of Oxford, he described three detailed cases of a “peculiar form of disease occurring mostly in young women, and characterized by extreme emaciation” [4]. Later, some physicians, seeking to trace the history of anorexia, have retrospectively diagnosed the conditions of female medieval saints as anorexia nervosa. These saints voluntarily abstained from eating any kind of food, consequentially leading to death. Fasting and virtuous self-abnegation have been attributed with the terms “holy anorexia” or “anorexia mirabilis”. However, they are extremely far from the real meaning of “anorexia nervosa”, because, on one hand, they are focused on seeking perfection and purity, and on the other hand they are from a distinct and an entirely different sociocultural context. The model of St. Catherine has been followed by medieval women who claimed to abstain from all food except the Eucharist, to prove devotion, the strength of the spirit, penitence, and pureness. Soon, the clergy identified the problem of recurrent holy fasting and responded with precise guidelines focused on good works rather than fasting for beatification. During the Renaissance, anorexia mirabilis seemed to disappear, only to reappear later as a form of protest, heretical, socially dangerous, and sometimes considered Satanically inspired [5].

Under a religious aspect, periodic fasting has been practiced by humans for centennials as a crucial spiritual purification pathway [6].

Aside from spiritual purposes, religious fasting has the power to affect physical health. The scientific community has focused on three different types of religious fasting [7]. The first is Islamic Ramadan, the holy month (28–30 days) for Muslims during which they refrain from eating, drinking, and smoking from sunrise (Sahur) to sunset (Iftar). Ramadan fasting is similar to Alternate-Day Fasting (ADF) because the 12 h fasting period is alternated with a 12 h feast period. It differs from ADF because drinking water is allowed during the 12 h fasting [8]. From a physiological point of view, Ramadan brings biochemical changes. The primary change is daily intermittent liver glycogen depletion and repletion. In the morning, carbohydrates are the main source of energy; more lately towards the afternoon, lipids become more important until fast breaking at sunset. This practice leads to changes in sleeping and activity patterns, circadian rhythms, and hormones release, including cortisol, insulin, leptin, ghrelin, growth hormone, prolactin, sex hormones, and adiponectin [9]. The second category of fasting includes the three principal fasting periods of Greek Orthodox Christianity (Nativity, Lent, and the Assumption). The diet consists of vegetables, plant-based foods such as legumes, fruits, nuts, bread; seafood and snails are moderately allowed, while fish and olive oil are permitted on defined days according to the specific fasting period [10]. As for the composition of Greek Orthodox Christian fasting, it does not appear as proper starvation, but rather as abstinence from certain types of food which brings nutritional and physical changes. A lower body mass is observed and an improvement in the lipid profile with reduction of total and LDL-cholesterol. While the intake of proteins, total fats, saturated fats, and trans-fatty acids decreases during fasting periods, carbohydrates and fibers appear to increase, which may demonstrate a change in serum lipids. Micronutrient amounts remain unchanged [11].

The third and last form is the Biblical-based Daniel Fasting. Like Greek Orthodox Christian fasting, the Daniel Fast may be considered as a form of dietary restriction, but it is stricter. It involves a 21-day ad libitum vegan food intake (inclusive of fruits, vegetables, whole grains, legumes, nuts, and seeds) with privation from animal products, preservatives, refined foods, white flour, additives, sweeteners, flavorings, caffeine, and alcohol. Bloomer et al. demonstrated that following Daniel Fasting for 21 days significantly reduces different metabolic and cardiovascular parameters and indexes, in particular systolic and diastolic blood pressure, total, LDL and HDL cholesterol, insulin, HOMA-IR, and C-reactive protein [12].

Historically, humans all over the globe have undergone through several periods of famine and forced fasting. War, imprisonment, and Nazi concentration camps are some examples. Contrary to the precedent types of religious fasting, forced fasting must be intended as a form of hunger and starvation. Usually, it leads to extensive malnourishment such as stunting, cachexia, and Kwashiorkor linked with poverty, food insecurity, and an increase of infections. Energy intake is severely low compared to metabolic expenditure, leading to weight loss. In particular, prisoners were affected by multiple pathologies linked to immune depletion and susceptibility to infections [13].

The Minnesota Starvation—Recovery Experiment and Starvation Study is an important clinical study performed at the University of Minnesota during the World War II period, November 1944 to December 1945 by Keys et al. The goal of the study was to examine starvation consequences in normal-weight people. After 6 months of caloric restriction intervention, subjects experienced a 25% loss of initial weight, and dramatic physical, psychological, and social changes. This research was significant in understanding eating disorders since some physical symptoms, and psychological and social behaviors, are similar to what was observed in patients with eating disorders. Among the physical modifications, the panorama of malnutrition symptoms were found, such as abdominal pain, difficulty in digestion, sleeping disorders, appendicular edema, tissue frailty, hair loss, sarcopenia, and increased risk of fractures [14]. It has to be considered that this was the first study on human starvation carried out in a tough social context prolonged for a long intervention time. Nowadays, studies on caloric restriction in humans are conducted for a shorter period and under ethical monitoring to ensure the safety and efficacy of the chosen protocol.

### 1.2. Fasting as a Choice for Longevity and Health Benefits

The act of fasting gained an increased focus in the scientific panorama thanks to several pieces of research developed around 30 years ago. The first studies referred to minor organisms and not directly to humans, because fasting and caloric restriction were considered tough interventions, combined with health risks if not adequately structured. Initially, studies on yeasts and murine models brought remarkably interesting results, later to be replaced in humans [15].

Some people, to be committed to their health, try adopting new habits as nutritional styles change. Nowadays, people are motivated by information from various sources: media, social networks, doctors, gyms, health coaches, and, simply, word of mouth and rumor. The accumulated information is not in line with scientific discoveries and safety protocols. Mere abstinence from food cannot result in efficacy if it is not well contextualized within a structured nutritional intervention. Fasting improves blood biomarkers for metabolic health, stress resistance, and suppresses inflammation. For example, most Westerners emulate their idols, picking up a fasting model that is supposed to help with losing and sustaining weight, keeping mentally sharp, and promoting longevity [16]. Most of the time they do not experience the suggested benefits because of an unbalanced diet [17].

In the light of the above, the goal of our paper is to examine the context in which “fasting” could be practiced, and the most important discoveries in fasting used in pathological conditions such as CDD [18]. Moreover, it aims to offer to clinical experts in Nutrition a specific guide to be consulted and personalized for each patient.

## 2. Fasting Response in Different Organisms

Fasting is the most extreme dietary restriction intervention because it requires the complete elimination of nutrients, but not water [19]. Organisms react differently to extended lack of food. Some become dormant, like yeast which enters a stationary phase; nematodes overcome a Dauer state, whereas bears and ground squirrels hibernate [20]. In mammals, organs like the liver and adipose tissue work as an energetic pool in the fasting and starvation period. According to the amount of fasting, length of time is variable and depends on species. At the same time, evolution in the fasting state leads to refine and ameliorate the metabolic, nervous, and endocrine system pathways in terms of performance of physical and mental activity [21].

The mechanisms of action in fasting were firstly understood in Saccharomyces cerevisiae yeast. A study conducted by Wei et al. in 2008 [22] demonstrated that removing glucose from the growing medium substrate and providing water only achieved the downregulation of the Tor–S6K and Ras–adenylate cyclase–PKA pathways. Normally, yeast’s chronological lifespan extension is regulated by serine/threonine kinase Rim15, whose activation is caused by deficiencies in Ras2, Tor1, and Sch9 in calorie restriction. This led to the consequent activation of the stress resistance transcription factors Msn2/4 and Gis1, which regulate many protective and metabolic genes [23]. Similarly, in the nematode *C. elegans*, transcription factor DAF-16 has the same role as the above-mentioned Msn2/4 and Gis1 in yeast [24]. Other findings in *C. elegans* identified the role of the GTPase RHEB-1 mechanism in extending lifespan [25]. In flies like Drosophila, intermittent food deprivation seems to affect longevity but not lifespan [26]. Murine models represent the most commonly used mammalian organism to explore the role of genes and processes associated with lifespan extension. In the absence of malnutrition, reduction in nutrient intake extends lifespan in many different species thanks to the mTORC1 inhibition [27]. Dietary restriction reduces mTORC1 activity in yeast, worms, fruit flies, and mice models, along with the reduction of IIS signaling [28]. Insulin/IGF-I signaling deficient mice report a delayed onset of fatal malignancies, increased insulin sensitivity, and a reduction in age-dependent cognitive impairment, including protection from Alzheimer’s disease (AD)-like associated pathologies [29]. Finally, in mice models, the first health benefit of caloric restriction is a lower incidence of cancer [30]. Not only caloric restriction in general, but specifically protein restriction (low protein diet), is associated with improving health span, increased lifespan, and inhibition of some neoplasms [31].

Among primates, an excellent model to investigate human aging is the Rhesus Monkey (*Macaca mulatta*) [32]. It shares about 93% sequence identity with the human genome, and studies have referred to anatomy, physiology, neurology, endocrinology, and immunology [33]. For this reason, non-human primates are considered important models for transferring pre-clinical research into clinical application. In non-human primates, the starting point of practicing caloric restriction is a key factor in determining the beneficial effect of aging. Proof in the literature has shown that caloric restriction may reduce the risk for age-related morbidity by more than two-fold. Like rodents, in monkeys the incidence of cancer is lower with caloric restriction. The tumor-suppressing capacity of caloric restriction is a constant conserved feature of mammalians [34,35]. Given the obvious link between rhesus monkeys and humans, it could be supposed that the beneficial effects of caloric restriction could also be observed in more evolved species.

### Metabolic and Biochemical Effects of Starvation in Humans

The above-mentioned beneficial effects of caloric restriction and fasting are known, but still under deeper investigation. One of the primary studies on humans was a two-year research conducted by Walford et al. inside an artificial and isolated structure called Biosphere 2. This was inspired by the Keys’ Minnesota study. Biosphere 2 was an opportunity to study the human responses to a prolonged two-year diet under carefully monitored conditions. The subjects studied consumed a low-calorie nutrient-dense diet rich in vegetables, fruits, nuts, grains, and legumes with small amounts of dairy, eggs, and meat. After a marked bodyweight reduction, they evaluated biochemical parameters concerning nutritional status that suggests a complex adaptive response linked to changes in calorie restriction [36].

In 2001 the National Institute of Aging of the US National Institutes of Health started longer-term studies to explore the age-related benefits and prolonged eligibility of CR in humans. The Comprehensive Assessment of Long-term Effects of Reducing Intake of Energy (CALERIE) trials are part of a research program aiming to test the effects of CR on longevity and aging biomarkers. Based on CALERIE phase 1, CALERIE phase 2 (CALERIE 2) was the largest study to date to assess sustained CR in healthy individuals without obesity and became the largest and most systematic examination of prolonged CR in humans. CALERIE 2 showed that moderate CR, 11.9% on average, induced improvements in aging-related biomarkers without negatively influencing psychological or behavioral outcomes [37].

Another large study of CR in humans was the Caloric Restriction with Optimal Nutrition (CRON study) where people practiced long-term severe CR with adequate intake of nutrients [38]. These subjects, who called themselves CRONies, were protected against abdominal obesity, T2DM, hypertension, dyslipidemia, inflammation, and CVD. The CRONies followed a daily diet poor in refined and processed foods with a high glycemic index and/or containing salt, trans-fatty acids, and dietary glycotoxins. Like the MD, CRONies consumed a wide variety of fresh vegetables, moderate intake of fruits, nuts, low-fat dairy products, egg whites, plant proteins (soy and wheat), fish, and lean meat. In CRONies, athletes who switched their protein intake from 1.6 g/kg body weight to around 1 g/kg resulted in a 25% reduction in serum IGF-1 concentration, suggesting that in humans protein intake is more important than calorie intake in modulating IGF-1 concentrations [39].

In literature, clinical and epidemiological studies demonstrated how fasting can delay the aging processes and associated diseases such as CDD. While energy restriction in humans is implied in aging control, oxidative damage to proteins, DNA, and lipids is a major factor that accelerates this [40]. Reactive Oxygen Species (ROS) are the result of the intracellular metabolism of cellular organelles like mitochondria, peroxisomes, and cytosolic enzyme systems, as well as several external agents that may trigger their production. Chemically speaking, ROS includes diverse species such as superoxide anions, hydroxyl radicals, and hydrogen peroxide [41]. The first two are extremely unstable, whereas others are largely diffused and longeval. Physiological regulation and homeostasis are modulated by a sophisticated enzymatic and non-enzymatic antioxidant defense system including catalase (CAT), superoxide dismutase (SOD), and glutathione peroxidase (GPx). The metabolic role of oxidants in cellular proliferation and host defense may be interrupted by lowering ROS levels below the homeostatic set point. Equally, aging, age-related diseases, and cell death can be determined by increasing ROS levels. For this reason, ROS levels must be included in a controlled range, where cells can be functional and responsive to stressors [42]. Caloric restriction administered on alternate days seems to reduce markers of protein-stress damage such as protein carboxyl in humans [43].

As discussed in this article, nutritional interventions of caloric restriction could be able to attenuate age-associated epigenetic alterations and could have a protective effect against concomitant cellular alterations, promoting longevity and health span. this promotes the maintenance of methylation and attenuates epigenetic drift [44]. During aging, genomic DNA methylation can occur, recognized as a biomarker of aging, and implicated in multiple common age-related diseases [44,45].

Several human studies have focused on potential perspectives on fasting in DNA damage and Insulin Growth Factor 1 (IGF-1) level [46,47]. Elevated circulating IGF-1 levels are associated with an increased risk of developing cancers. People with genetically severe IGF-1 deficiency caused by the deficiency of growth hormone receptor rarely develop cancer because their cells are protected from oxidative stress-induced DNA damage. Moreover, DNA damage highly promotes programmed cell death. Hence, fasting may protect from cancer by enhancing the death of pre-cancerous cells and reducing cellular and DNA damage [48].

It is known that caloric restriction may improve inflammatory and autoimmune diseases [49].

Monocytes are producers of pro-inflammatory cytokines and play a key role in the induction and maintenance of inflammation. Circulating monocytes are increased in overweight and obese patients. Caloric restriction has been shown to modulate the reduction of peripheral pro-inflammatory cells which leads to a total improved inflammatory profile. Short-term fasting or caloric restriction seems to decrease susceptibility to pathological inflammatory disease [50,51]. Fasting processes may reduce the size of the circulating monocytes pool and modify metabolic activity and gene expression patterns working on prognosis and amelioration of inflammatory and auto-immune disorders. However, a low-grade inflammation does not always have to be intended as a negative response. Hence, monocyte emergency mobilization is essential during acute infectious inflammation and tissue repair [49].

In humans, macro-autophagy mediated by autophagosome has been investigated in various neurodegenerative diseases including AD. A study conducted with ultrastructural analysis of post mortem human brains revealed that AD increased autophagosomes in dystrophic neurites [52]. Macro-autophagy is the main actor for generating Amyloid beta (Aβ) in the cytoplasm [53]. Macro-autophagy has a more direct relationship with some degenerative diseases and fasting may control autophagic pathways through chemicals or vectors. Hence, nutritional starvation induces macro-autophagy in neurons, but this induction is not sufficient to destroy a high amount of Aβ in AD-associated pathological conditions. For this reason, fasting must be included in a personalized combination of drugs and dietetic therapy [54].

Caloric Restriction (CR) inhibits the mechanistic target of rapamycin (mTOR) activity [55,56]. mTOR is a kinase composed of two complexes with different physiological functions, mTORC1 and mTORC2 [57,58]. Pharmacological or genetic inhibition of mTORC1 signaling results in increased lifespan. High activity of mTORC1 is a major impulse for aging, while its suppression contributes to the many benefits of CR, including lifetime extension [59].

mTORC1 and mTORC2 are regulated by nutrients, hormones, and growth factors. In humans, mTORC1 activity reduces during fasting and increases after feeding. Studies reported that mTORC1 activity significantly oscillates during the day hours, under the control of internal circadian clocks. Notably, CR and biological circadian clocks are interconnected. CR modulates circadian rhythms in behavior, chromatin modifications, and gene expression, and some related proteins are involved [60].

## 3. Fasting Physiology and Models

### 3.1. Physiology of Fasting

Physiologically, fasting consists of the catabolism of lipids, proteins, and carbohydrates to maintain blood glucose levels in a normal range. After an overnight fast, the most dominant source of plasma glucose is the liver glycogen. Glycogen stores are depleted within about 48 h. After that period, gluconeogenesis precursors like gluconeogenic amino acids, pyruvate, and lactate come mainly from the muscle. Glycerol is another gluconeogenic precursor released by adipose tissue after the hydrolysis of triglycerides. During fasting, the uptake of gluconeogenic amino acids by the liver tends to rise. At the same time, ketone bodies are released gradually into the plasma, and they may be used as a biomarker of lipolysis and fatty acid β-oxidation capacity in tissues. Most of these changes are caused by low plasma insulin levels, increases in catecholamines and glucagon [61], and amelioration of insulin sensitivity [62].

### 3.2. Types of Fasting

From ancient times up to nowadays, different types of fasting have appeared along with new scientific discoveries. Now, it is possible to classify fasting diets according to the pattern of eating window, the potential physiological process, and health benefits.

A basic definition of Time Restricted Feeding (TRF) is limiting the time of eating to a specific number of hours each day [1]. Intermittent Fasting (IF) is a prescription of a diet approach that alternates between a period of eating and fasting, usually not more than 24 h [63]. Inside this category, we found Alternate Day Fasting (ADF) [64] and Modified Alternate Day Fasting (MADF) [65]. Finally, Prolonged Fasting (PF) is fasting for an extended period, usually, more than 2 days [65] (See Table 1 for details).

### 3.3. Metabolic and Physiological Responses in Animal and Human Models

#### 3.3.1. Effects of TRF

Extending the daily fasting duration in animal models, TRF promotes the mobilization of free fatty acids (FFA), and increases fat oxidation and the production of ketones [69]. It appears to alter the gut microbiome to one that is less obesogenic [69]. When animals are fed a high-fat diet (HF diet), TRF allows the restoration of the diurnal variation in several families of bacteria that are involved in nutrient absorption [76]. Lastly, TRF without a marked calorie restriction can promote and maintain the synchronization of the metabolic and behavioral circadian clock [77].

Concerning humans, in patients who restricted their Energy Intake (EI) to ~20% reduction in estimating daily caloric intake, a loss of body weight (maintained for 1 yr), an improvement in sleep satisfaction, less hunger at bedtime, and increased energy levels were observed [71,78], along with a significant reduction in body fat percentage measured by bioimpedance [79].

Among nutritional factors, TRF has emerged as an innovative strategy to prevent and treat mental health disorders, sleep disturbances, and cognitive impairment [80]. Feeding/fasting timing manipulation has emerged as an innovative strategy to contrast and treat cognitive decline. TRF may be positively associated with cognitive status, and thus exert plausible effects on brain health. Studies reveal a reduction in levels of Brain-Derived Neurotrophic Factor (BDNF), a biomarker linked to age-related cognitive deficit. Fasting may enhance synaptic plasticity, neurogenesis, and neuroprotection especially by an increase in BDNF, which also affects neural precursor cells (NPC) and play a role in spatial pattern separation, a fundamental domain of learning and memory [81].

#### 3.3.2. IF: Effects of ADF and MADF

In obese mice, the ADF regimen with an HF diet results in weight loss, improved blood glucose control and daily fluctuations in selected physiological and biochemical parameters [82]. Glucose control is improved on fasting days with the use of ADF in a genetic model of obesity in the face of minimal weight loss [83]. ADF could significantly inhibit Type 2 Diabetes (T2D) induced insulin resistance and obesity, promote insulin signaling, reduce inflammation, and promote glycogen synthesis and lipid metabolism. It possibly depends on mitochondrial biosynthesis and energy metabolism [84].

Concerning humans, ADF prolonged for 22 days leads to weight loss and other benefits such as amelioration of metabolic profile, especially for blood test, blood pressure, and inflammation [85]. A strict ADF seems to improve markers of general health in healthy, middle-aged humans while causing a 37% calorie reduction on average. ADF improved cardiovascular markers, reduced fat mass (particularly trunk fat), improving fat-to-lean ratio, and increased β-hydroxybutyrate, even on non-fasting days. On fasting days, the pro-aging amino-acid methionine, among others, was periodically depleted, while polyunsaturated fatty acids were elevated. Long-term ADF reduced levels of sICAM-1 (an age-associated inflammatory marker), low-density lipoprotein (LDL), and the metabolic regulator triiodothyronine [86]. ADF diet performed on non-alcoholic fatty liver disease (NAFLD) patients appeared to achieve a reduction in body weight and improvement of dyslipidemia within a relatively short period (4 to 12 weeks) [87].

Similar to ADF, in mice MADF decreases fat cell size in the inguinal and epididymal region [88], plasma total cholesterol, TG, free fatty acid concentrations, leptin, and resistin levels. MADF modified adipose tissue physiology, in terms of body fat distribution, TG metabolism, and adipokines, in a way that may prevent coronary heart disease [89].

In humans, a reduction of body weight, waist circumference, fasting plasma glucose [67], total cholesterol, TG, LDL, and blood pressure was observed [90]. MADF may improve several key biomarkers of Coronary Artery Disease (CAD) risk, such as obesity, waist circumference, fat mass, systolic and diastolic blood pressure. It also seems to be able to modify serum lipid profile [65].

#### 3.3.3. Effects of PF

To deal with prolonged fasting, animals undergo important acute physiological adjustments such as hypothalamic epigenetic changes and long-lasting metabolic adaptations in mice [91]. Fasting induced changes in hormone balance, body weight, metabolism, hepatic enzymes, cardiovascular parameters, body temperature, and toxicological responses [92].

In humans, during PF catabolic and anabolic metabolome, non-esterified fatty acids, glycerol, ketone bodies, and amino-acids on urinary excretion are produced [61]. Reduced inflammatory status is an important mechanism. Sustained 40–50% reductions in circulating concentrations of the pro-inflammatory cytokines TNF-α and CRP are revealed, plus, relative to ad libitum, CR improved total white blood cell, lymphocyte, and monocyte counts [93,94]. Fasting for 3 or more days leads to a 30% or more decrease in circulating glucose and insulin, as well as rapid decline in IGF-1 levels and an increase in one of the principal IGF-1-inhibiting proteins, IGFBP1 [28,95]. Recently, increased circulating adiponectin concentrations were observed [96].

## 4. Is Fasting Safe? Body Composition and Nutritional Status Emendation

The main objective of a dietetic plan is to preserve lean mass and ameliorate the body composition, considering the age range of the population examined [97]. For example, sarcopenia is a remarkable concern of the aging population. Sarcopenia is defined as the loss of skeletal muscle mass and its function [98]. Correlation between the fasting model and sarcopenia may be reflected as a short period of the starvation protocol, with an adequate per-meal and daily protein intake, according to the lean mass [99].

Despite the general belief that fasting may cause muscle loss, human clinical trials show the opposite. This concept is reinforced by gluconeogenesis from protein that starts around 24 h after the fasting starts. The hormonal adaptation to fasting also protects the lean body mass physiologically [100].

During fasting, the whole body weight is reduced, particularly in terms of fat mass and total body water. Short periods of an engineered diet which mimics fasting have been revealed to preserve lean body mass and bone mass while promoting fat storage depletion, mostly located in trunk fat [101].

A study that analyses TRF and Resistance Training (RT) shows that TRF did not compromise improvements in muscular strength and did not lead to decrements in lean soft tissue. Despite this, TRF may have altered hypertrophic adaptations to RT. Regarding body composition changes during TRF, subjects with normal body fat at baseline experienced increases in lean tissue. Contrarily, pre-obese or obese individuals tended to lose lean soft tissue. Focusing on muscular strength, TRF does not appear to be detrimental to muscular improvements, and the magnitude of improvements in muscular strength and endurance was equal to or similar to subjects following a normal diet [102].

While most fasting studies point out weight and fat mass decreases, it is important to define specific guidelines for fasting and non-fasting days to enhance adherence, minimize dropout rates from the interventions and maximize body composition improvement [66].

Hence, to evaluate the best fasting protocol, clinicians must consider and verify the nutritional status of patients. This nutritional status assessment has to be performed with care and precision. It consists of physiological, pathological, and dietetic anamnesis, anthropometry, plus different methods of body composition evaluation.

Concerning body composition, compromised nutritional status may be associated with the occurrence of complications such as cachexia, micronutrient depletion, sarcopenia, osteopenia, and osteoporosis [103].

## 5. Guidelines

### 5.1. Inclusion and Exclusion Criteria

Fasting is a dietetic protocol that may be followed by most patients but there are some limitations according to specific features. Firstly, children and adolescents must be excluded from any type of fasting model, as it could be psychologically and physiologically dangerous. The same attention should be pointed out for pregnant or breastfeeding women, and elderly people over 75 years old. These groups need precise energetic and nutritional requirements, and it would not be appropriate to eliminate essential nutrients, even if for a short period. In the case of active infection, risk of repetitive infection, fever, cough, diarrhea, or persistent immune depletion, fasting is not suggested, given that genetic DNA mutations may compromise alternative glucose-source oxidation. Generally, fasting is not permitted in underweight status for Body Mass Index (BMI). However, in an underweight condition for BMI, but with normal condition of Free Fat Mass (FFM), defined with specific parameters of nutritional status as Appendicular Skeletal Muscle Mass Index (ASMMI) [104,105] and Body Cellular Mass Index (BCMI) [106,107], controlled fasting intervention should be used to reduce oxidative stress and promote cellular clean-up. Although some studies suggest that fasting may be helpful for people with diabetes in improving insulin sensitivity and glucose control along with modest decreases in body weight [21], the American Diabetes Association does not recommend fasting as a technique for diabetes management [108]. Finally, patients with a diagnosis of eating disorders are not ideal candidates for the fasting model. There is no consistent evidence supporting scientific fasting in ED; moreover, religious fasting, like Ramadan, could act as a trigger or exacerbator for ED in adolescents [109].

According to the latest data on pharmaceutical prescription in Italy, in 2019 seven citizens out of ten received at least one drug prescription, with agender difference, men 62% and women 71% [110]. Considering this scenario, the nature of the drug prescribed must be considered before evaluating fasting. If people take a multi-drug prescription, clinicians must consider the combination of drug effect and influence of caloric restriction on the patient. For example, people taking antihypertensives need to monitor the blood pressure and heart rate throughout the fasting period. Other kinds of pathological conditions must be assessed and monitored before following a fasting model and its application.

### 5.2. General Recommendations

Since fasting consists of a particular dietetic approach, it is recommended that clinicians guide patients before it begins.

Principally, it is suggested to avoid processed and hypercaloric meals because the metabolic response from binging to fasting would be violent. Soft drinks and alcoholic beverages should be avoided in the immediate time before fasting. Concerning caffeine, there are two different theories. The first supports the use of caffeine during fasting days, thanks to some proven benefits. In this case, the amount of caffeine/day ingested must follow the Recommended Dietary Allowance of 200 mg/day which does not raise concerns for the adult and healthy population [111]. This quantity corresponds to three cups of coffee per day. Other sources of caffeine (e.g., caffeinated beverages) must be excluded. Caffeine can decrease body weight through the increase in thermogenesis and fat oxidation via the inhibition of phosphodiesterase, which degrades intracellular cyclic Adenosine Monophosphate, and the inhibition of negative effects of adenosine on increased noradrenaline release [112]. Moreover, it is scientifically proven that coffee can modulate appetite, acting on gastric emptying [113], and caffeine consumption has been associated with the prevention of cognitive decline, and reduced risk of neurological and neurodegenerative diseases such as Parkinson’s and Alzheimer [114]. Caffeine controls microglia-mediated neuroinflammatory response associated with most neurodegenerative conditions thanks to the action of adenosine receptor, particularly A2A receptor [115]. Finally, coffee consumption is associated with a reduced risk of conditions that share low-grade inflammation as their physio-pathological basis [116]. The second theory refuses the consumption of caffeine during fasting days. Fasting purists drink only water because they believe that drinking any other kind of beverage can break the fast or influence it negatively [117].

For this reason, it would be better to prepare the body for fasting with a more balanced diet, personalized and rich in plant-based foods and water. The best approach is a Mediterranean-based plan plentiful in flavonoids, fibers, and micronutrients [118]. For people who have irregular eating habits, clinicians suggest starting to eat regularly with an appropriate macronutrient repartition and avoiding any processed food or beverage for about a week before fasting.

### 5.3. Behavioral Recommendations during Fasting Days

Although there is no proper consensus list of behavioral recommendations during fasting days, one of the goals of this paper is to propose this. It is suggested that clinicians explain fasting features and any undesirable effects to patients. First, during fasting, it is recommended to rest and to avoid strenuous and physical activities, both planned and spontaneous. Fasting could be intended as a holistic approach and the only physical activity allowed is soft gymnastics or yoga [119]. A different condition applies for athletes, to whom it is suggested to train at relatively low intensities (not at high-intensity levels) when fasting, to ensure that they recover adequately to optimize performances in competitive events [120]. Concerning spontaneous activity, it is recommended to avoid driving for a long time and undertaking domestic tasks. For people who work in heavy jobs, it would be preferable to follow fasting during day/days off. Environmental temperature can affect body physiology during fasting. Euthermia should be preferred. Data in the literature showed that seasonal variation in Heart Rate and Blood Pressure, including systolic blood pressure (SBP) and diastolic blood pressure (DBP), have been linked to seasonal changes in outdoor temperature. Cold weather can stress the body and affect the heart in several ways. Lower temperatures can cause blood vessels to narrow, and the heart must work harder to move blood throughout the body. Cold weather can also affect the heart by increasing blood pressure and heart rate [121]. Higher outdoor temperatures or exposure to hot temperatures, like sauna or hammam baths, may cause low blood pressure and high sweating rate with consequent loss of mineral salts. The latter may lead to dehydration and syncope [122].

Concerning smoking, it is recommended to avoid it in general. Studies have shown an increased smoke exposure during fasting. It seems that humans in a food-deprived state tend to increase drug intake. Smoking behavior also changes in response to fasting with increased puff volume and carbon monoxide (CO) exposure [123]. Cigarette smoking alters physiological aspects like blood pressure, heart rate, inflammation, oxidative stress, toxic elements such as heavy metals, and tar accumulation in the lungs. This induces an increased risk of cancer, heart disease, stroke, lung diseases, diabetes, and chronic obstructive pulmonary disease (COPD), which includes emphysema and chronic bronchitis, respiratory problems, tuberculosis, certain eye diseases, and problems of the immune system, including rheumatoid arthritis [124]. Thus, we strongly believe that smoking during fasting days may counteract the benefits at all levels.

### 5.4. Eating Recommendation before and after Fasting Days

Before and after fasting days, whatever the fasting length, it is extremely important to prepare the body. It is usually recommended to follow a regular and balanced diet. We suggest a High Nutritional Quality Diet (HNQD) based on anti-inflammatory and Mediterranean guidelines. Food intake should be divided into five meals a day: breakfast, lunch, dinner and two snacks. The Mediterranean model is based on whole grains, fresh fruits, and vegetables (five portions per day), legumes, nuts, and daily use of olive oil. Plant proteins and fresh fish are usually preferred to red meat. Processed foods are excluded from the diet. A small amount (125 g) of red wine is allowed once a day. Diet should be personalized according to the patient, considering a caloric restriction for those who are overweight and obese, and an isocaloric diet for normal-weight. We recommend a daily macronutrient distribution as follow: 55% of total kcal/day of carbohydrates (sugars < 10%), 20% of total kcal/day of protein (>50% of vegetable derivation), <25% of total kcal/day of lipids (on total daily energy intake: saturated fat <10%, 6–10% polyunsaturated fatty acids (PUFA), n-6/n-3 PUFA ratio of 3:1, 15% of monounsaturated fatty acids (MUFA); <1% trans-fatty acids) and 25 g of fiber [125]. Protein intake is a crucial element to ensure the maintenance of Lean Mass (LM). Diet should be balanced starting from daily protein amount, with a protein intake of 2 g/Kg of total LM, according to Colica et al. [126,127]. Starting with and maintaining an adequate LM is compulsory to reach all the benefits of the fasting state in terms of body composition. Maintaining a correct nutrition status is crucial, especially in a period when the immune system is stimulated and involved in internal reset [128,129]. Water intake is also important. We recommend a total water intake of 1.8 L/24 h for all individuals to satisfy body needs and balance [130]. Hydration is one of the elements that regulate both circulatory and lymphatic systems. The lymphatic system is the body’s own waste removal service and a major component of the circulatory system. It is twice as large as the arterial system, and it comprises a network of tissues and vessels that transport and dispose of lymphatic waste and other fluids. It plays a central role in our immune response: lymphocytes (white blood cells) originate from and are transported into the lymphatic system to fight off diseases and infections. An impaired lymphatic system will lead to a weakened immune response, increasing the risk of getting sick [131].

## 6. Recommendation in Patients with Diabetes Mellitus

Despite its potential for disease prevention and treatment, prolonged fasting is difficult to implement in human subjects under therapies.

Caloric restriction and weight loss influence, in a positive way, the course of Type 2 Diabetes (T2D). A loss of 5% of body weight or more may reduce glycated hemoglobin (HbA1c), lipoprotein levels, and blood pressure [132].

In human type 1 diabetes (T1D) pancreatic islets, fasting conditions reduce PKA and mTOR activity and induce Sox2 and Ngn3 expression and insulin production. Prolonged fasting promotes the reprogramming of pancreatic cells to restore insulin generation in islets from T1D patients and reverses both T1D and T2D phenotypes in mouse models [133].

The most diverse evidence on fasting in humans included T2D patients who were treated with lifestyle advice only or also with a dose of metformin. However, T2D patients who use other types of anti-diabetic medication, especially insulin, might be at risk of developing hypoglycemia while using this medication in combination with the prolonged forms of fasting [134].

The primary risk with a fasting diet is hypoglycemia in patients who are on antidiabetic medications. Specifically, insulin (both prandial and basal) and sulfonylureas (including the short-acting meglitinides) are associated with a high risk of hypoglycemia. Other antidiabetic therapies when used as monotherapy or in combination therapy without insulin or sulfonylureas are rarely associated with hypoglycemia. The risk is therefore considerably less yet still probable.

Among the most important recommendation for people with diabetes, it is strongly recommended to maintain adequate protein intake in those days when they are eating and, depending on how many days the patient is fasting, it may be necessary to take vitamin and/or mineral supplements. Hydration must be taken under control.

People with diabetes and in comorbidity with other chronic diseases may increase the risk of experiencing many of the dehydration linked adverse events: dizziness, nausea, insomnia, syncope, falls, migraine headache, weakness that limits daily activities, and excessive hunger pangs. We recommend encouraging good hydration during any fasting regimen. Drinking water, including replacing fluids that normally would be consumed in foods, is important for people of all ages who are participating in fasting protocol [62].

All the already mentioned exclusion criteria are compulsory for consideration and reinforcement in the case of diabetes.

## 7. Recommendation in Patients with Cardiovascular Diseases

In light of the previous considerations on fasting and biochemical–physiological effects on the cardiovascular system, our position is to evaluate each patient as a single clinical case, considering the variability of CVD features from patient to patient. However, we strongly recommend avoiding fasting protocol in case of recent Myocardial Infarction (MI). This latter is important in order to make any possible fasting regimen experience as safe as possible in a particular condition. Hence, in the case of elderly people with CVD clinical history, we suggest considering stricter inclusion criteria at around 65yo instead of 75yo.

## 8. Recommendation for Patients with Cancer

The concept of a frail patient includes the idea that declining physiological reserves and function leads to higher susceptibility. Among the most important spectrum of frailties, we can focus on Cancer and Eating Disorders (ED) diagnosis.

Clinical studies of protocols that mimic fasting in patients undergoing chemotherapy support its feasibility and overall safety. In a small trial, prolonged fasting helped patients maintain their quality of life during chemotherapy, and reduced fatigue [135]. Particularly, in patients with hormone-receptor-positive breast cancer receiving estrogen therapy, cycles of diet that mimic fasting cause metabolic changes analogous to those observed in preclinical studies, including reduced levels of insulin, leptin, and IGF1. Our recommendation for clinicians is to wait for more evidence, because these results support further clinical studies on fasting as an adjuvant to hormone therapy in various types of breast cancer [136].

## 9. Conclusions

Through this work, we supply clinicians with a how to guide on fasting. Starting from the fasting model of choice, we turn to the main recommendations to observe during the fast, and which dietetic approach to prescribe between starvation and feeding states, to enhance and maintain fasting benefits in non-fasting periods. The literature contains plenty of studies describing fasting models. Given this, we nevertheless observed a lack in the guidelines approaching which diet therapy to follow before and after starvation. Through our fasting guide, we provide accurate indications, a sort of “posology” of fasting, to which clinicians may refer to elaborate specific nutritional strategies. While we are the first to gather all the features of a fasting protocol in this article, prospective studies must focus on fasting models in different pathological conditions.

## Figures and Tables

**Table 1:** Different types of Fasting.

	Time Restricted Feeding (TRF)	Intermittent Fasting (IF)		Prolonged Fasting (PF)
		Alternate Day Fasting (ADF)	Modified Alternate Day Fasting (MADF)	
Definition	This is an eating pattern in which the food intake is restricted to a time window of 8–12 h or less every day [1].	This form of IF involves fasting every other day or on certain days of the week. Ad libitum caloric intake is followed on non-fasting days [66].	This is a form of IF, similar to ADF, with a severe and specific caloric restriction on fasting days. During fasting days, the caloric intake consists of 15–25% of the dietary needs. Ad libitum diet is followed on non-fasting days [67].	PF consists in fasting for an extended period, from 4 to 7 days. It has been less commonly studied for longer periods in humans [68].
Characteristics	Limiting the eating duration may be an effective strategy to reduce the overall caloric intake. It does not necessarily have to involve caloric restriction [69].	Starving one day, feasting the next. Only during the fasting days is a caloric restriction expected [66].	Restriction days are non-consecutive and include only a small introduction of food. During non-fasting days the intake of food is at leisure [65].	During consecutive fasting days usually only water is permitted [70].
Commonly Practiced Method	16/8: feeding window of 8 h/day in which it is allow to consume food and 16 h of fasting [71]. 12/12: feeding and fasting windows last the same. This is claimed to be the simplest type of TRF to improve health and to maintain weight [72].	5/2: This is the most common example of ADF. Calories are severely restricted for 2 days (preferably non-consecutive), and then normal eating occurs for the other 5 days in the week [73].	5/2: 15%–25% of Total Daily Energy expenditure (TDEE) is suggested during 2 non-consecutive fasting days a week. Ad libitum food intake for the resting 5 days [74].	No commonly practices methods are defined. Periods of deliberate fasting with restrictions on intake of solid food are practiced [75].

## Abbreviations

CR	Caloric Restriction
CDD	Chronic Degenerative Diseases
ADF	Alternate Day Fasting
LDL-Cholesterol	Low Density Lipoprotein Cholesterol
HDL	High Density Lipoprotein
TOR-S6k	Target of Rapamycin-S6 Kinase
PKA	Protein Kinase A
Ras2	Rat Sarcoma 2
TOR1	Target of Rapamicin 1
Sch9	Serin/Threonin-Protein kinase
Msn/4	heterodimeric zinc-finger transcription factor
Gis1	Transcriptional activator/repressor **GIS1**
GTPase RHEB-1	Regulatory Protein G of mTOR
mTORC1	Mechanistic Target of Rapamicyn Complex 1
IIS	insulin/IGF (insulin-like growth factor)-like signalling
IGF1	Insulin Growth Factor 1
AD	Alzheimer Disease
DNA	Deoxy Ribonucleic Acid
ROS	Reactive Oxygen Species
CAT	Catalase
SOD	Superoxide Dismutase
GPx	Glutathione Peroxidase
Aβ	Amiloide Beta
mTor	Mechanistic Target of Rapamicyn
mTORC2	Mechanistic Target of Rapamicyn Complex 2
TRF	Time Restricted Feeding
IF	Intermittent Fasting
MADF	Modified Alternate Day Fasting
HF diet	High Fat diet
TG	Triglycerides
PF	Prolonged Fasting
BDNF	Brain-Derived Neurotrophic Factor
NPC	Neural Precursor Cells
BMI	Body Mass Index
FFM	Free Fat Mass
RT	Resistance Training
ASMMI	Appendicular Skeletal Muscle Mass Index
BCMI	Body Cellular Mass Index
SBP	Systolic Blood Pressure
DBP	Diastolic Blood Pressure
CO	Carbon Monoxide
COPD	Chronic Obstructive Pulmonary Disease
HNQD	High Nutritional Quality Diet
MUFA	Mono-Unsaturated Fatty Acids
PUFA	Poly Unsaturated Fatty Acids
LM	Lean Mass
T2D	Type 2 Diabetes
HbA1c	Glycated Haemoglobin
T1D	Type 1 Diabetes
Sox2	SRY (sex determining region Y)-box 2
Ngn3	Neurogenin3
MI	Myocardial Infarction
